# Crystallization of the β-Form of Polypropylene from the Melt with Reduced Entanglement of Macromolecules

**DOI:** 10.3390/polym16121710

**Published:** 2024-06-15

**Authors:** Justyna Krajenta, Andrzej Pawlak

**Affiliations:** Centre of Molecular and Macromolecular Studies, Polish Academy of Science, 90-363 Lodz, Poland; justyna.krajenta@cbmm.lodz.pl

**Keywords:** polypropylene, crystallization, entanglements, spherulites, β-form crystals, equilibrium melting temperature, crystallization regimes

## Abstract

The influence of decreasing the entanglement density of macromolecules on the crystallization of the β-form of polypropylene was investigated. Polypropylene with seven times less entanglement was obtained from a solution in xylene, and its properties were compared with those of fully entangled polypropylene. To obtain a high β-phase content, the polymer was nucleated using calcium pimelate. In non-isothermal crystallization studies, accelerated growth of β-crystals was found, increasing the crystallization temperature. Also, the isothermal crystallization was fastest in the nucleated, partially disentangled polypropylene. Increased growth rate of spherulites and enhanced nucleation activity in the presence of more mobile macromolecules were responsible for the high rate of melt conversion to crystals in the disentangled polypropylene. It was also observed that the equilibrium melting temperature of β-crystals is lower after disentangling macromolecules. Better conditions for crystal building after reduction of entanglements resulted in enhanced crystallization according to regime II.

## 1. Introduction

Isotactic polypropylene represents polymorphic materials, i.e., polymers able to crystallize in various crystallographic forms, called α, β, γ, and smectic modifications [1,2,3,4]. The most common form is the monoclinic α-form. Compared to the α-form, the β-form occurs much less frequently during bulk crystallization [5].

The β-phase of polypropylene is metastable and easily transforms into the stable α-phase. It melts at a temperature of 155 °C, much lower than α-phase with a melting temperature of 170 °C [6]. The melting temperatures, as usual for polymers, are much lower than the equilibrium melting temperature, *T_m_*°. *T_m_*° values for the α-form are given in the range between 184 and 220 °C. The equilibrium melting temperatures of the β-form were found in the range of 170–200 °C [7,8,9]. Precise measurements by Juhasz et al. [9] gave the value *T_m_*° = 209 °C when the non-linear approach of Hoffman–Weeks was applied and *T_m_*° = 177 °C using Hoffman–Weeks linear extrapolation. The values of the heat of fusion measured by different authors also differ, although there is an agreement that the heat of fusion of the α-phase is greater than the heat of fusion of the β-phase. According to Monasse and Haudin [10] and Varga and Garzo [11], the heat of fusion of the α-phase is 146 J/g and that of β-phase is 113 J/g. Li et al. [7] proposed use values of 178 J/g for the α-phase and 170 J/g for the β-phase. However, Jiang et al. [12] claimed that the commonly used heat of fusion of 100% crystalline α-iPP, β-iPP, and isotactic polypropylene (iPP) are 177.0, 168.5, and 209 J/g, respectively.

The formation of β-crystals is observed in a certain temperature range, between 100–105 and 140–141 °C for ordinary iPP [6,13] or between 90 °C and 133 °C for the low tacticity iPP [14]. In this temperature range, the growth rate of β-crystals is faster than that of α-crystals, which is attributed to a difference in the free energy of addition molecular stems at the (110) growth face of the β-crystals [6,15]. Outside this range, the α-form grows more easily, and the metastable β-form, if nucleated, readily converts to the α-form [5]. It was found that β-crystals are formed when the melt cooling rate is lower than 50 K/s [15], but the efficiency of β-phase formation decreases when cooling is too slow [16]. For example, during non-isothermal crystallization at rates of 20–80 K/min, the β-phase content exceeded 60%, while at 5 K/min, it was only 15% [17].

It is possible to increase the content of the β-form by crystallization in a special way, i.e., by applying melt shear [18] or by crystallizing the polymer in a temperature gradient [8,19]. However, the most common is the addition of a specific nucleating agent to the polymer, which not only increases the number of nuclei but also promotes the formation of crystals of appropriate modification [20,21,22,23]. Among the nucleating agents used are calcium pimelate [14,21,24], γ-quinacridon, or *N*,*N*′-dicyclohexylterephthalamide [25]. The addition of the above nucleants also resulted in an increase in the non-isothermal crystallization temperature, a shortening of the isothermal crystallization time, and a reduction in the size of the formed spherulites [3,14,20,21,22,23,24,25].

During the crystallization of polypropylene, a spherulitic structure is usually formed [13]. A characteristic feature of α-form spherulites is that they are composed of both radial and tangential lamellae, while β-form spherulites have a classic radial arrangement of parallel lamellae. Early studies on the formation of β-spherulites by Padden and Keith [16] showed that the morphology of spherulites depends on the crystallization temperature and negative birefringence radial or banded (ringed) spherulites could grow. β-form spherulites observed with a polarizing microscope are brighter than α-form spherulites [26]. When β-nucleants were used, hedritic structures were found in the early stages of growth of spherulites of this variety [13]. Norton and Keller [27] suggest the formation of radial β-spherulites for crystallization below 142 °C and nucleation of ringed spherulites in the range of 126 °C to 132 °C. Similarly to the α-polypropylene, a change in the formation of a new layer on the crystal surface at a certain temperature, i.e., the occurrence of crystallization regimes, was observed for the β-phase. The available data concerns the Regime II–Regime III transition, which was observed in the temperature range of 123–130 °C [28], at 133 °C [13], or at 132.5–139 °C, depending on the brand of iPP [29].

The course of the polymer crystallization depends on the ability to transport macromolecules to the crystallization sites. The mobility of a macromolecule is limited by its entanglements with other macromolecules. Entanglements of macromolecules occur in the melt, solution, and amorphous phases of solid polymers. It is assumed that typically processed polymers have an equilibrium entanglement density, with a different value for each type of polymer. This density is characterized by giving the average molecular weight between the entanglements, *M_e_*. For example, for isotactic polypropylene, measurements in various laboratories gave *M_e_* values in the range of 5500–9990 g/mol. [30,31,32,33].

Methods are available to reduce the entanglement density of macromolecules. In laboratory scale, the solvent method with freezing is most often used. It is known that with greater dilution of the polymer solution, the number of contacts between macromolecules, which take the shape of a coil, decreases. In a very dilute solution, even complete separation of the macromolecule coils is achievable. If the polymer solution is suddenly frozen, e.g., using liquid nitrogen, the entanglement state of macromolecules will be preserved. After removing the solvent, e.g., by sublimation, a solidified polymer with a lower density of macromolecule entanglements is obtained. The choice of solvent depends on the polymer. Polypropylene is usually disentangled at high temperatures in xylene, decalin, or trichlorobenzene [34]. There are variants of this method, e.g., stabilization of the disentangled state in PP can be achieved by crystallization while cooling the hot solution. In recent years, studies have been carried out on the crystallization of a number of polymers after entanglement reduction, e.g., polylactide, poly(ethylene oxide), polyethylene, and isotactic polystyrene [35].

Crystallization of polypropylene from the melt was also carried out [36,37,38,39,40]. These studies concerned a partially disentangled polymer, in which almost only the α-form grew. Under non-isothermal conditions, the disentangled sample had a crystallization temperature 2–5 °C higher than the entangled sample [38]. The transformation of the melt into a solid state was also faster when entanglements were reduced [41]. It was observed in isothermal studies that after disentangling, the growth rate of spherulites increases [36,37,39]. After reducing entanglements, a shift in the transition temperature between regimes II and III by 3–4 °C was observed [38]. An important practical information from the rheological tests of partially disentangled PP was that even at high temperatures of 200–210 °C, the re-entanglement of macromolecules did not occur very quickly, which means that during crystallization studies there remains at least 10–15 min of time available for the necessary melting of the polymer [41].

So far, no studies have been carried out on the crystallization of β-form polypropylene from the melt with a reduced density of macromolecular entanglements. We prepared appropriately modified polypropylene and performed both isothermal and non-isothermal crystallization tests. Crystallization studies carried out using differential scanning calorimetry and polarization microscopy with a heating stage were complemented by X-ray scattering studies. Interesting results showing the influence of the entanglement density of macromolecules are described below.

## 2. Materials and Methods

The examined polymer was isotactic polypropylene (iPP) Novolen 1100 N produced by BASF (Ludwigshafen, Germany). According to the producer, it had a molecular mass of 250 kg/mol, a *M_w_/M_n_* ratio of 5.0, a density of 0.936 g/cm^3^, and a melt flow rate of 11 g/10 min (2.16 kg, 230 °C). The nucleant was calcium pimelate, which was synthesized from pimelic acid and calcium oxide according to the procedure described by Lezak and Bartczak [42,43].

Partially disentangled polypropylene was prepared by dissolving 0.5 wt.% of iPP Novolen 1100 N in xylene at 135 °C. After mixing the polymer with the solvent for 1 h, the solution temperature was lowered at a rate of 25 °C/h. When the temperature reached 80 °C, gel formation was observed, which was visible as the turbidity of the solution. After further cooling to *T* = 40 °C, the gel was removed from the flask and dried. As a result of the process, polypropylene powder with reduced entanglement was obtained. Rheological measurements showed that the molecular mass between the entanglements increased to *M_e_* = 19,100 g/mol [41], almost twice the initial *M_e_* = 9900 g/mol. In a three-dimensional network of macromolecules, this means approximately seven times fewer entanglements. In the remainder of the text, for simplicity, a partially disentangled polymer will be referred to as disentangled.

Using a Zamak–Mercator (Krakow, Poland) co-rotating twin-screw mini-extruder, disentangled polypropylene was mixed with the calcium pimelate nucleant. The following mixing conditions were used: temperature 185 °C, time 2 min, screw rotation 100 rpm. The nucleant concentration was 0.2 wt.%. Samples were prepared from both disentangled and entangled polypropylene. The crystallization of these samples was compared with the crystallization of non-nucleated entangled and non-nucleated disentangled polypropylene. The list of prepared samples along with the abbreviations used is provided in Table 1.

A TA DSC Q-20 (TA Instruments, New Castle, DE, USA) differential scanning calorimeter (DSC) was used to study non-isothermal crystallization. The samples weighing 7–9 mg were heated from 0 °C to 190 °C at a rate of 10 °C/min, held at this temperature for 1 min, then cooled at a rate of 10 °C/min to 0 °C and heated again at a rate of 10 °C/min to 190 °C. Some studies were also carried out at higher cooling rates, i.e., 30 and 50 °C/min. The main parameters determined from non-isothermal DSC experiments were crystallization temperature, melting point, and crystallinity.

DSC has also been used to analyze thermal effects during isothermal crystallization. During the isothermal tests, the samples were heated to the temperature of 220 °C, held there for 1 min, and then cooled at a rate of 10 °C/min until the desired crystallization temperature, in the range of 126–145 °C, was achieved. This temperature was maintained as long as changes in heat flow were visible. The heat flow data were used to calculate the progress of the melt transformation into crystals with crystallization time and to describe the crystallization process using the Avrami approach.

Crystallization of the PPd_02 or PPi_02 sample at the selected temperature and then heating at a rate of 10 °C/min to measure the melting temperature was the procedure used to determine the equilibrium melting temperature.

The spherulitic crystallization in isothermal conditions was examined using a Linkam THMS 600 (Linkam Scientific Instruments, Redhill, UK) hot stage attached to the Nikon (Nikon Corporation, Tokyo, Japan) polarizing light microscope, which was equipped with a CCTV camera connected to the computer. For observations, the piece of PP was sandwiched between two microscopic cover glasses situated on the stage, melted for 1 min at 220 °C, and compressed into a 15 μm thick film. The molten film was cooled at a rate of 10 °C/min to the temperature of isothermal crystallization and kept at this temperature until the end of the observations. The temperature of crystallization was in the range 128–145 °C. During the experiment, the expansion of spherulites was recorded with time, and from the position of the boundaries was determined the growth rate. At least the growth of 10 spherulites was measured.

The contents of the β- and α-phases in samples after controlled crystallization were determined by the wide-angle X-ray scattering technique (WAXS). Two-dimensional WAXS images were recorded using a Pilatus 100 K (Dectris AG, Baden-Daettwil, Switzerland) detector coupled to a CuKα X-ray source (sealed tube, operating at 30 kV and 50 mA, Philips (Eidhoven, The Netherlands)). Based on these images, scattering intensity profiles as a function of scattering angle were determined using ImageJ 1.54 (National Institutes of Health, Bethesda, MA, USA) software. The scattering peak intensities were used to determine the *K* parameter from the Turner–Jones equation [44], which characterizes the share of β-crystals in the total amount of crystals.

## 3. Results and Discussion

### 3.1. Non-Isothermal Crystallization

**Figure 1:**
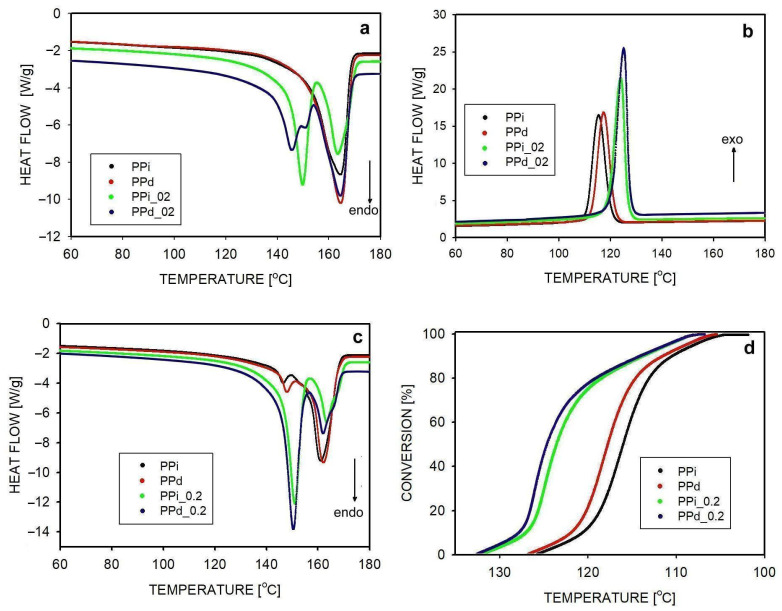
Non-isothermal properties of polypropylenes studied by DSC with a heating/cooling rate of 10 °C/min: (**a**) first heating, (**b**) cooling, (**c**) second heating, and (**d**) conversion of melt into crystals during crystallization.

The course of non-isothermal crystallization examined using DSC is shown in Figure 1. Polypropylenes without additional nucleation and those containing 0.2% nucleant were tested with a cooling/heating rate of 10 °C/min. The first heating curves (Figure 1a) show the melting of samples that crystallized under uncontrolled conditions at the end of extrusion. In non-nucleated polypropylene, both fully entangled and partially disentangled, a melting peak is visible at 165 °C. It corresponds to the melting of the α-phase. The presence of the β-phase is not visible. This is different in the case of melting of nucleated samples, where two melting peaks are visible with maximums at temperatures of 146–148 °C and 165 °C. The first of these peaks corresponds to the melting of the β-phase. For sample PPd_02, the β-phase peak is divided into two. The higher temperature peak is a result of β to β′ recrystallization [1].

The crystallization of polypropylenes at a constant melt cooling rate is shown in Figure 1b. It started at the earliest, at a temperature of 133 °C, in sample PPd_02, then in PPi_02 (132 °C), PPd (127 °C), and at the latest in PPi, at 126 °C. The order in which the peak maxima occur is the same, and their values are given in Table 2. The dynamics of the crystallization process are most visible in Figure 1d. It shows the conversion of the melt into the crystalline phase as the temperature changes. Crystallization occurs most rapidly in the polymer, where there are more crystallization nuclei and in which macromolecules have greater mobility, which favors their transport to the places of crystal growth.

Figure 1c shows the melting process after crystallization. In all samples, two melting peaks are visible, related to the melting of the α- and β-forms that were formed during crystallization. The melting maximum of the β-phase is observed in the temperature range 147–150 °C, and, as shown in Table 2, it is higher for nucleated polypropylenes. The α-phase shows a maximum melting peak at higher temperatures, in the range of 161–1646 °C. Also, in this case, slightly higher temperatures correspond to the melting of the nucleated polymers. A higher melting point indicates, in accordance with the Gibbs–Thomson rule [45], that thicker crystals melt, which is consistent with their earlier formation and slightly better growth conditions.

Table 2 also shows the heats of crystallization and melting. The results for the first heating are related to the previous processing and therefore only show that a significant amount of crystalline phase was formed in all samples. Much more important are the results of measurements of the heat of crystallization and heat of fusion during the second heating. As can be seen from Table 2, regardless of the differences in the crystallization temperature, the crystallization heat values are similar, in the range of 84–89 J/g. The corresponding heats of fusion have similar values, as was expected. Slightly higher values may result from the transformation of the β-phase into the α-phase, having a higher heat of fusion at 100% crystallinity. Since separate peaks are visible in the nucleated samples during melting, it was possible to estimate the share of the β-phase in the heat of fusion. For the PPi_02 sample, the heat of melting of the β-phase was 67 J/g, and for the PPd_02 sample also 67 J/g, which indicates a significant share of this phase in both tested samples. If the heat of fusion of 177 J/g for the fully crystalline α-phase and 168.5 J/g for the 100% crystalline β-phase [12] is taken into account in the calculations, the degrees of crystallinity of these phases in both nucleated samples are similar and are respectively 14% and 39%. This means that the β-phase content is approximately 0.73 of the total crystalline phases.

In our previous studies, we showed that the disentangling of macromolecules promotes faster non-isothermal crystallization of non-nucleated PP, in which crystals of the α-form were formed almost exclusively [38]. Since, as can be seen from Figure 1b, crystallization in conditions where the β-form dominates is the fastest in PPd_02, this means that also for β-phase, reducing the entanglement of macromolecules and facilitating their diffusion accelerates the growth of β-crystals in non-isothermal conditions.

Similar calculations are difficult to perform for the heat of melting of non-nucleated PPi and PPd samples, where the small melting peak of the β-phase is located on the slope of the melting peak of the α-phase. Approximate estimates indicate that the β-phase content in these samples does not exceed 2%.

These results can be compared with commonly performed measurements of β-phase content using the X-ray WAXS method. In the case of polypropylene, it is difficult to directly determine the share of both crystal forms from the X-ray diffractogram because some signals originating from diffraction on crystal planes overlap. Therefore, the equation proposed by Turner–Jones is commonly used to determine the share of the β-form [25,44]. This equation has the following form:

*K* = *I_beta_*_300_/(*I_beta_*_300_ + *I_alfa_*_110_ + *I_alfa_*_040_ + *I_alfa_*_130_) (1)

where *I_beta_*_300_ is the scattering intensity (i.e., peak height) from the (300) plane of the β-form. Moreover, *I_alfa_*_110_, *I_alfa_*_040_, and *I_alfa_*_130_ represent scattering on the (110), (040), and (130) planes of the α-form, respectively. When determining the intensity, the amorphous phase was subtracted.

**Figure 2:**
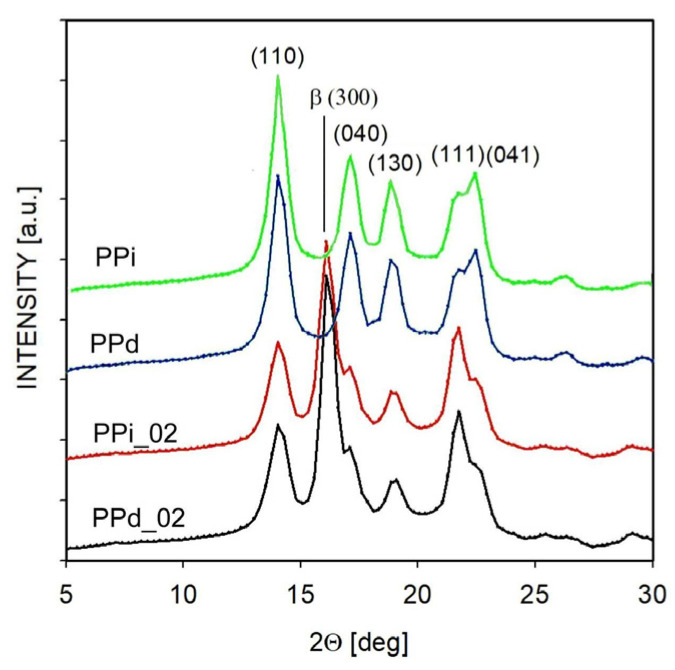
X-ray diffractograms for selected polymers, recorded after the non-isothermal crystallization.

Figure 2 shows typical diffractograms of the tested samples, with an indication of the scattering on the planes used for calculations from Equation (1). For samples PPi_02 and PPd_02, the scattering peak on the β-phase dominates, while in the case of PPi and PPd, the signal from the β-phase is practically invisible.

Table 3 shows the content of the β-phase, determined for the tested materials in accordance with Equation (1). As can be seen, with crystallization at a rate of 10 °C/min, the β-form content for both nucleated polypropylenes is 0.76–0.77, and for the non-nucleated PPi and PPd it is close to zero. These results agree well with estimates from DSC measurements.

For samples containing nucleants, non-isothermal crystallization tests were additionally performed at higher rates of temperature changes, i.e., 30 °C/min and 50 °C/min. The results are presented in Table 2. Our previous work on crystallization [38] examined partially disentangled and entangled polypropylene, in which no additional nucleation was used. It was observed that at higher cooling rates there was a difference in the crystallization temperature between partially disentangled and entangled PP, and the temperature values decreased, i.e., crystallization occurred later. For example, at a cooling rate of 50 °C/min, the crystallization temperature was 109.7 °C for disentangled and 103.1 °C for entangled PP. The second observation reported in this work was a significant difference in the heat of crystallization, which was lower by up to 8 J/g for entangled polypropylene when the cooling rate was 50 °C/min. The above results are not included in Table 2, because they have been published, and we now focus on nucleated, β-rich samples.

The results in Table 2 show that in the case of nucleated polypropylenes at higher cooling rates (30 °C/min and 50 °C/min), crystallization occurs at increasingly lower temperatures. In these polypropylenes, as in a non-nucleated polypropylene, there is still a difference in melting temperature between entangled and disentangled polypropylene. The decrease in *T_c_* with faster cooling results from the fact that a certain time is necessary to activate nucleation. A large number of active nuclei introduced into the samples marginalized the effect of easier movement of macromolecules and caused the difference in crystallization temperatures between PPd_02 and PPi_02 to be smaller than previously mentioned for PPd and PPi. Although the crystal growth stage is dominated by the mobility of macromolecules, greater in disentangled polymers [46,47], the time available for crystallization is short, and therefore the difference in the heat of crystallization between partially disentangled and entangled polypropylene is not very large.

Analyzing the data in Table 2 regarding both melting processes (i.e., during the first and the second heating) of the nucleated samples, heated at rates of 30 °C/min and 50 °C/min, it should be noted that there are no two separate peaks for both crystalline phases, as in the case of heating at a rate of 10 °C/min, but one broad peak, with a maximum at a temperature intermediate to the melting temperatures of both phases. The position of this peak changes towards higher temperatures as the heating rate increases. There is a slight difference in melting point between entangled and partially disentangled PP. The higher heat of melting than the corresponding heat of crystallization can again be attributed to the β/α transition.

The analysis of X-ray diffractograms allowed the determination of the share of the β-phase in samples crystallized non-isothermally at cooling rates of 30 °C/min and 50 °C/min. The results are included in Table 3, supplemented with data for previously unmeasured PPi and PPd. Table 3 shows that in the nucleated samples at high crystallization rates, the β-phase still dominates, even slightly increasing in content to 0.83–0.84 at a cooling rate of 50 °C/min. In the case of non-nucleated PPi and PPd, a higher cooling rate during crystallization does not result in an increase in the content of the β-phase, which remains at the level of 2–5% of the total crystalline phase.

### 3.2. Isothermal Crystallization

**Figure 3:**
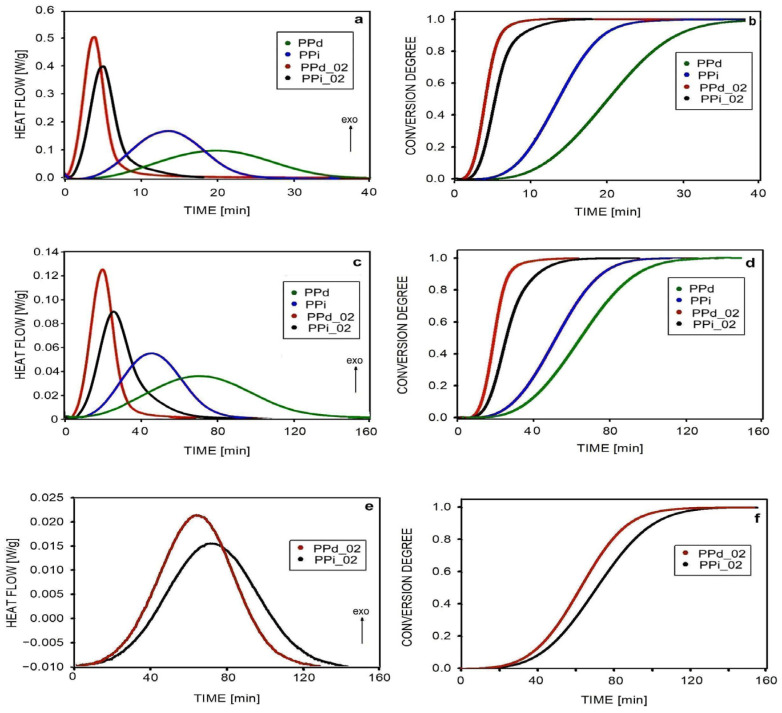
Changes in heat flow with time for samples crystallized at temperatures 135 °C (**a**), 140 °C (**c**), 145 °C (**e**), and the conversion of the melt into the crystalline phase during measurements at temperatures 135 °C (**b**), 140 °C (**d**), and 145 °C (**f**).

Isothermal crystallization studies began with a general analysis of the crystallization process using DSC, conducting experiments in the temperature range of 130–145 °C. Figure 3 shows changes in heat flow with crystallization time at selected temperatures of 135, 140, and 145 °C and the conversion of the melt into the crystalline phase at these temperatures.

Figure 3a shows the course of crystallization of samples at the temperature of 135 °C. The crystallization process began immediately after the samples reached the set temperature. However, the dynamics of crystallization, characterized by heat flow, were very different in non-nucleated and nucleated polypropylene. In non-nucleated PPd and PPi, the process took a long time, 35 and 25 min, respectively. Curves showing more precisely the dynamics of melt conversion are shown in Figure 3b. The slower crystallization of PPd resulted from the removal of some of the nuclei during polymer disentangling. Increasing the number of nuclei by adding 0.2 wt.% of the nucleant accelerated the crystallization process. Of the two nucleated materials, PPd_02 crystallized faster. The number of nuclei in PPd_02 and PPi_02 was similar, and as previous and current (see below) studies on polypropylene have shown, the crystals in the disentangled polymer grow faster. Therefore, the whole crystallization was completed the fastest in PPd_02 (after 10 min) and a little later in PPi_02 (after 14 min).

The heat changes due to the crystallization of samples at 140 °C are shown in Figure 3c. The course of conversion of the melt into crystals at this temperature is shown in Figure 3d. Of course, a higher crystallization temperature means a longer time during which this process takes place. At a temperature of 140 °C, the crystallization sequence of the samples was almost the same as at a temperature of 135 °C. The PPd sample still crystallized the slowest because the higher crystal growth rate did not compensate for the loss of some nuclei during disentangling. At a temperature of 140 °C, crystallization was completed the fastest for the PPd_02 sample, where the high density of nuclei and the higher rate of crystal growth led to the fastest conversion of the melt into the crystalline phase.

Figure 3e shows the course of crystallization at 145 °C. Due to the slow crystallization of non-nucleated samples at this temperature, measurements were performed only for samples containing 0.2 wt.% of the nucleating agent. At this temperature, the beginning of the crystallization process was visible 8 min after reaching the temperature. The kinetics of crystalline phase formation were similar in PPi_02 and PPd_02 (see Figure 3f), although partially disentangled polypropylene crystallized slightly faster. Full crystallization of both materials required approximately 2 h.

Microscopic observations in polarized light of the samples after crystallization and calculations of the *K* factor using the WAXS showed that only single β-spherulites were present in the PPd and PPi samples, and it was difficult to distinguish the peak (300) from the β-phase in the diffractograms. However, in the case of both nucleated samples, the diffraction on the (300) plane of the β-phase dominated the diffractogram. This indicated a significant contribution of the β-phase. These comments apply to crystallization at all three temperatures, i.e., 135 °C, 140 °C, and 145 °C. The values of the K coefficients, showing the share of the beta phase in the total crystalline phase, determined for nucleated samples crystallized at three temperatures are presented in Table 4.

The content of the β-phase in the nucleated samples depended on the crystallization temperature and was the highest (*K* = 0.70–0.74) at 135 °C. At a crystallization temperature of 140 °C, more than half of the crystals still had the β-phase structure. According to literature reports [6,13], this is the upper temperature limit for the formation of β-spherulites in the absence of specific nucleation. In the tested samples PPd_02 and PPi_02, the β-fraction was formed at a temperature of 145 °C, but its share was minor. As in the samples crystallized at lower temperatures, more β-phase was formed in the entangled polymer than in the disentangled. This was probably due to the greater number of nuclei present in PPi than in PPd before additional nucleation, part of them giving β-crystals.

Microscopic observations showed that a spherulitic structure is formed in the tested polypropylenes during isothermal crystallization, and the majority of spherulites begin their growth at the same time. With such a course of crystallization, it can be described according to the approach proposed by Avrami [12,48,49]. The Avrami equation has the following form:

1 − *X* = exp(−*zt^n^*) (2)

where *X* is volume fraction of crystallized material; *t* is the time; and *z* and *n* are coefficients characterizing the nucleation process. The *z* coefficient is described by the following equation:

*z* = 4π*NG*^3^/3 (3)

where *N* is density of nucleation and *G* is growth rate of spherulites. Equation (2) can be expressed as follows:

ln[−ln(1 − *X*)] = *n* ln *t* + ln *z* (4)

The data used to calculate the conversion of the melt into crystals (Figure 3) were also used for calculations according to Equation (4). Linear conversion ranges over time were used to determine the Avrami coefficients. The values of *n* and *z* are presented in Table 5. Since the tested samples contained both α- and β-spherulites, which differ in their growth rate and share in the total, the interpretation of the results was possible to a limited extent [50].

The values of the *n* coefficient range from 2.9 to 3.8 and indicate three-dimensional growth of spherulites with heterogeneous nucleation, initiated in a short period of time, which is consistent with optical microscopic observations. According to Mandelkern [51], the value of the *n* coefficient in the range of 3 < *n* < 4 indicates three-dimensional heterogeneous nucleation; the value in the range of 2 < *n* < 3 indicates a two-dimensional heterogeneous nucleation. The tendency is visible in the calculations (see Table 5) that lower *n* values are obtained for a lower crystallization temperature. The reason for this is the increase in the share of instantaneous nucleation compared to sporadic nucleation [52].

The values of the *n* coefficient at 135 °C are lower for disentangled PPs, which shows some influence of disentangling. In the case of PPd *n* is below 3. The exact reason for this is not sure. Isothermal crystallization was carried out using DSC, so it cannot be excluded from a more intensive nucleation on the contact surface of the melt with the DSC cell, as a result of which the growing spherulites have a less regular shape, which affects the dimensionality of the sample. Another possibility is some participation of non-spherical structures such as hedrites [53], which were sometimes observed by us using microscopy in the early stages of crystallization.

Table 5 shows that there are significant differences in the *z* values. Spherulite growth rate measurements are discussed in detail in the next part of the text. They showed that at a temperature of 135 °C, β-spherulites grow 1.22–1.28 times faster than α-spherulites, depending on whether the polymer was entangled or disentangled. β-spherulites and α-spherulites grow simultaneously inside the volume samples. To simplify the calculations according to Avrami, one average value for the spherulites growth rate in the material, *G*′, can be assumed and calculated according to the following equation:

*G′* = (1 − *K*) × *G*_α_ + *K* × *G*_β_ (5)

where *G*_α_ and *G*_β_ are growth rates of α-spherulites and β-spherulites, respectively.

The calculated *G*′ values are presented in Table 6. With these values, it is possible to estimate the nucleation density in the tested samples using Equation (3). The results of the spherulite nucleation density calculations are presented in Table 6.

The nucleation density decreases with increasing temperature, approximately one order for every five degrees. The external nucleation of the samples affected the total number of active nuclei, which increased by an order or two, depending on the temperature. At both crystallization temperatures, i.e., 135 °C and 140 °C, there were fewer nuclei in the non-nucleated PPd sample than in the non-nucleated PPi sample, which is most likely due to the removal of some of the nuclei during the disentangling of polypropylene in the solution.

**Figure 4:**
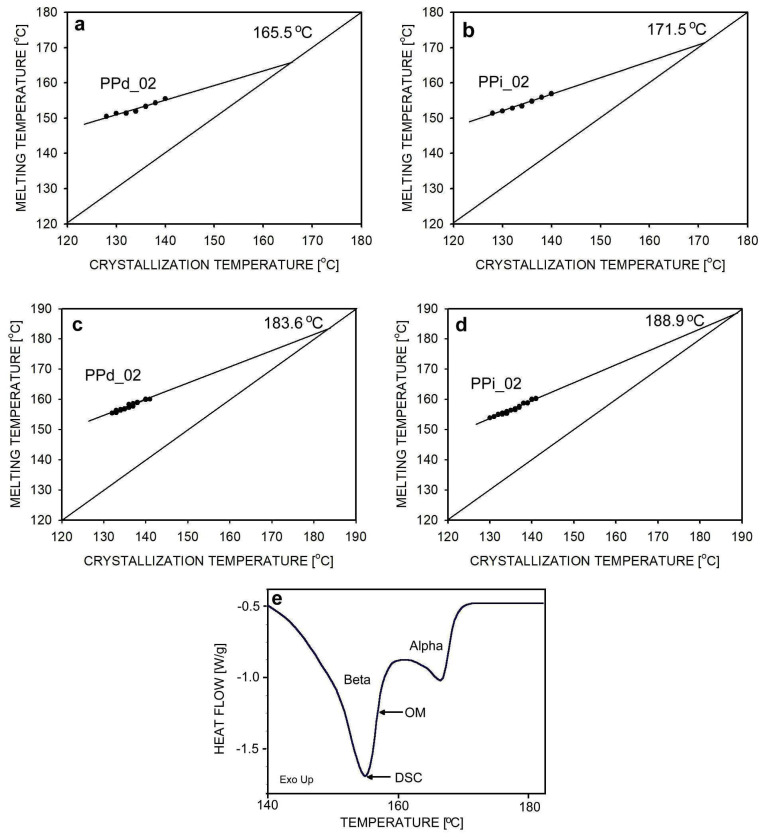
Determination of the equilibrium melting temperature from DSC measurements (**a**,**b**) and microscopic observations (**c**,**d**) for nucleated polypropylene. Also shown is the thermogram for the PPi_02 sample melted after crystallization at 136 °C (**e**). The equilibrium melting points determined by both methods, i.e., DSC and OM, are marked.

During the isothermal crystallization of polymers, a linear relationship between the crystallization temperature and the melting temperature is observed. This relationship is the basis of the Hoffman–Weeks method for determining the equilibrium melting temperature, *T_m_°*. Figure 4 shows the relationship between the melting temperature and the crystallization temperature for the β-form of polypropylene. Measurements were made using two methods, i.e., using a DSC apparatus and using a heating stage connected to a polarizing light microscope. In the first case, after crystallization of the PPd_02 or PPi_02 at the selected temperature, the sample was heated at a rate of 10 °C/min, which allowed for the observation of melting. The thermogram of melting showed two peaks related to the melting of the β- and α-phases. The position of the maximum of the β-peak was used for calculations. An example thermogram for the PPi_02 sample is shown in Figure 4e.

For studies using a polarizing microscope, the same crystallization and melting procedure was used as for studies using DSC. The melting of several selected spherulites was observed, and the temperature at which the spherulites were no longer visible was considered the melting temperature. The temperature determined by the second method was higher than the temperature determined by the first method. For example, for the PPi_02 sample crystallized at 136 °C, a temperature of 154.8 °C was recorded from DSC tests and 156.8 °C from microscopic examination. The arrows in Figure 4e show the position of these temperatures in the thermogram. Apart from a different choice of the melting moment, i.e., the peak maximum in DSC and the disappearance of spherulites in the microscopic image, some shift in the temperature measured by both devices due to technical reasons cannot be ruled out.

After measuring melting temperatures for a series of crystallization temperatures in the range of 128–140 °C, a straight line describing the experimental results was determined using the linear regression method. Then, its intersection with the *T_m_* = *T_c_* line was found, and the intersection point indicated the equilibrium melting temperature. The experimental relationships *T_m_* = *f*(*T_c_*) and the determined values of the equilibrium melting temperature are shown in Figure 4a–d.

Before discussing the obtained *T_m_°* values, we briefly present the limitations of the Hoffmann–Weeks method. The equilibrium melting temperature *T_m_°* is an important parameter in the description of crystallization. It is the melting temperature of a crystal of infinite size, equal to its crystallization temperature. The undercooling, i.e., the difference between the equilibrium melting temperature and the crystallization temperature of a real crystal, is proportional to the thermodynamic driving force for crystallization [54]. There is no direct method for measuring the equilibrium melting temperature, and the Hoffman–Weeks method is one of the two most commonly used [55]. The disadvantage of the method is that the *T_m_°* temperature is determined on the basis of several points located at a large temperature distance from the determined *T_m_°* value. Additionally, it is assumed that the *T_m_*-*T_c_* relationship is linear for the entire *T_c_* range and that isothermal crystal thickening, increasing *T_m_*, is not significant. If DSC results are used, the analysis is based on the melting of medium-sized crystals without taking into account the crystal thickness distribution.

For all these reasons, one might get the impression that the Hoffman–Weeks method is not useful. Strobl [56] analyzed the *T_m_*-*d* and *T_c_*-*d* relationships, where *d* is the crystal thickness. He found that there is an intersection of these two linear relationships, but at a point where the thickness *d* is not infinite but much smaller, so it cannot be the point determining *T_m_°*. The explanation was that the *T_c_*-*d* relationship is not linear at low undercoolings. Departure from linearity is also proposed in the literature when attempting to modify the original Hoffman–Weeks approach. Marrand and co-workers [9,57] proposed the use of nonlinear extrapolation of *T_m_*-*T_c_* data. This approximation leads to a higher *T_m_*° value than that obtained by linear extrapolation.

We used a simpler linear approximation to determine *T_m_°* because the goal was not to find the most likely equilibrium melting temperature value but to see if there is a difference between entangled and disentangled polypropylenes. For this purpose, *T_m_°* was determined in the same way for disentangled and entangled PP. The crystals from the disentangled polymer grew slightly earlier (see Figure 3), so they may have been slightly thicker due to the longer available annealing time, and therefore their *T_m_* may have also been higher. This would mean that the *T_m_°* for disentangled PP is overestimated, and therefore the difference in *T_m_°* for both polypropylenes may be even greater than observed.

In both experiments, the equilibrium melting temperature *T_m_°* for the PPd_02 sample with limited entanglements was 5.3–6.0 °C lower than the entangled PPi_02 sample. This is a large difference, indicating the beneficial effect of disentanglement on the size and perfection of the β-phase crystals formed at the selected temperature of isothermal crystallization. A shift in the equilibrium melting temperature, but much smaller, was previously observed in studies of poly(ethylene oxide) by Krajenta et al. [58]. The large difference in the values of the equilibrium melting temperature measured for the same polypropylene by two methods results from the described differences in the selection of melting temperature values for calculations. The equilibrium melting temperature determined for the β-phase of entangled polypropylene agrees with those reported in the literature [7,8,9].

**Figure 5:**
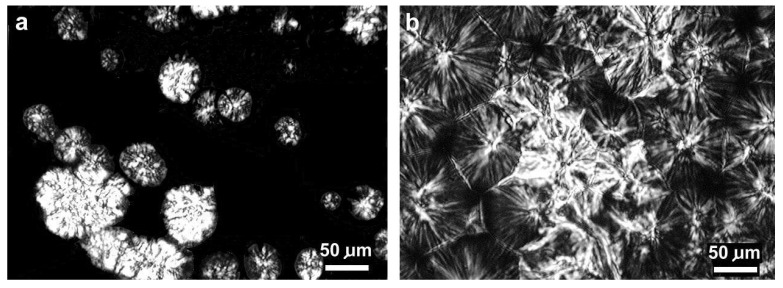
Isothermally-crystallized growing spherulites in the PPd_02 sample at 132 °C (**a**). The spherulitic structure in the PPi_02 sample formed after crystallization at 126 °C (**b**).

Observations using a polarizing microscope confirmed that spherulites are formed during crystallization in all tested samples. The photograph in Figure 5a shows the early stage of formation of the spherulitic structure in PPd_02. Because β-spherulites are characterized by greater birefringence than α-spherulites, they are visible as brighter objects. Figure 5b shows a fully formed spherulitic structure. Many spherulites have similar sizes and straight fragments of the boundaries, which indicates nucleation during the same period of time.

**Figure 6:**
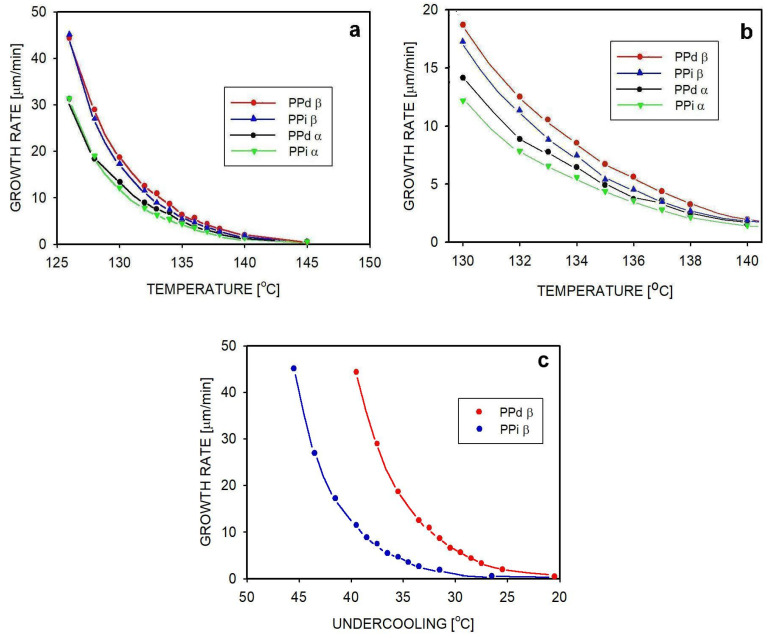
Spherulite growth rate as a function of crystallization temperature (**a**). Abbreviations in the figure: PPd β—β-spherulites in the PPd_02 sample; PPi β—β-spherulites in the PPi_02 sample; PPd α—α-spherulites in the PPd_02 sample; PPi α—α-spherulites in the PPi_02 sample. Part (**b**) shows the temperature range of the largest differences in the growth rate of spherulites between the tested samples. Part (**c**) shows the dependence of the growth rate of β-spherulites on undercooling. Lines on the graphs are shown for better visualization only.

During crystallization at a selected temperature, the growth rate of spherulites can be determined from the location of the spherulite boundaries as a function of time. Such measurements were performed in the temperature range of 126–145 °C for at least 10 spherulites of each tested material at each temperature. The average values of the spherulite growth rate at certain temperatures are presented in Table 7, and the curves describing changes in the spherulite growth rate as a function of time are shown in Figure 6. Due to the radial direction of growth of the lamellas forming the spherulite skeleton, the growth rate of the spherulite is also the growth rate of lamellar crystals.

Both in the entangled and disentangled polymer at temperatures below 145 °C, β-spherulites grew faster than α-spherulites. Moreover, at temperatures of 128–140 °C, β-spherulites in the disentangled polypropylene grew faster than in the entangled one. A similar phenomenon was previously observed for α-spherulites. The reason for the faster growth of spherulites in samples with limited entanglements is the easier transport of macromolecules to the surface of the growing crystals [46,47]. At high temperatures, the effect is no longer visible. Macromolecules, even entangled ones, have high mobility at these temperatures, so increasing this mobility by disentangling is no longer so important.

The data in Table 7 show that at the lowest crystallization temperature, i.e., 126 °C, the growth rates for the β-phase in entangled and disentangled iPP are similar. This might suggest that the degree of entanglement is no longer important. Figure 6c shows the dependences of the growth rate of spherulites not as a function of the crystallization temperature but as a function of the undercooling, calculated on the basis of equilibrium temperatures of 165.5 °C and 171.5 °C (see Figure 4). At the same undercooling, except for the smallest undercoolings measured, the growth of β-spherulites in the disentangled polymer occurred much faster than in the entangled polymer. This confirms the role of constraints from other macromolecules in the movement of a single macromolecule to the crystal growth sites, and thus the influence of entanglements on the crystallization rate.

According to the classical theory of crystallization by Hoffman and Lauritzen, the crystal growth rate, *G*, is controlled by two factors, i.e., the transport factor and the driving force factor, both of which depend differently on temperature [59,60].

The dependence of *G* on the crystallization temperature can be described by the following equation:

(6) $$G\left(T\right)=G_{o}exp\left[-\frac{\Delta E}{R\left(T-T_{ref}\right)}\right]exp\left[-\frac{K_{g}}{T\;\Delta T}\right]$$

where *G_o_* is a pre-exponential factor, depending on the molecular mass; Δ*E* is the activation energy of the transport of chain segments to the crystallization site (i.e., activation energy of reptation); *R* is gas constant; *T* is crystallization temperature; *T_ref_* is a reference temperature, below the glass transition, when a free volume is close to zero; *K_g_* is a constant depending on the regime of crystallization. Moreover, Δ*T* = *T_m_*° − *T*, where *T_m_*° is the equilibrium melting temperature. Equation (6) can be presented in the following logarithmic form:

log *G* + Δ*E*/[2.3 × *R*(*T* − *T_ref_*] = *f*(1/*T* Δ*T*) (7)

In the analysis according to Equation (7) of the growth of β-phase spherulites, we used the previously determined equilibrium melting temperatures of 165.5 °C and 171.5 °C. Other values used for calculations were Δ*E* = 6270 J/mol, *R* = 8.31 J/(mol × K), and *T_ref_* = 231.2 K [29,58,61]. The relationship described by Equation (7) has a linear character, with the slopes of the sectors changing at characteristic temperatures, defining the ranges of crystallization regimes.

**Figure 7:**
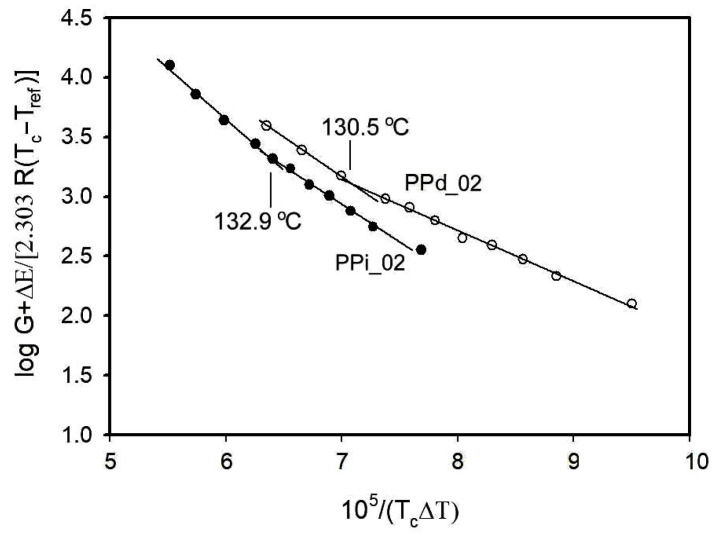
Results of measurements of the transition temperature between regimes II and III.

Figure 7 shows how the temperatures of transition from regime II to regime III were determined for β-phase crystallization in partially disentangled and equilibrium-entangled polypropylene. Temperature values obtained were 130.5 °C for disentangled PP and 132.9 °C for entangled PP. The last value agrees with the previously measured by Varga et al. [1,13].

According to Hoffman’s theory, the crystal formation process is different depending on the temperature regime and is more regular in regime II than in regime III. The lower transition temperature measured for PPd_02 means that in the temperature range of 130.5–132.9 °C, crystals are formed in the disentangled polypropylene according to the mechanism of regime II, while the crystals grow in the entangled polypropylene in the way predicted by the theory for regime III. The change in the regime boundary indicates the beneficial effect of partial disentanglement of macromolecules on the crystal formation mechanism, leading to greater regularity of structure with fewer defects. The shift in the temperature boundary between regimes II and III was previously observed for isotactic polypropylene, containing almost exclusively α-phase [38], and for poly(ethylene oxide) [58].

## 4. Conclusions

Previously conducted research on the crystallization of polypropylene, in which almost exclusively the α-form was formed, showed that limitations in the mobility of macromolecules in the melt due to the presence of other macromolecules influence the crystallization. Since, after external nucleation, it was possible to obtain a high content of the β-phase in crystallized polypropylene, and it has been known that under usual crystallization conditions, β-spherulites grow faster than α-spherulites, research was undertaken on the influence of the degree of entanglement of macromolecules on the crystallization in the β-form.

During non-isothermal crystallization, it was observed that the crystallization of samples, in which about 80% of the β-crystals were formed, occurred at a higher temperature and faster (conversion of the melt into the β-phase) when the polymer was disentangled. The heat of crystallization was slightly higher for disentangled polypropylene. The differences described above resulted from easier transport of less entangled macromolecules to the crystal growth sites. This accelerated the crystal growth process but did not help nucleation much because the time needed for additional nucleation was limited.

The isothermal crystallization was also examined, starting with a DSC analysis of the whole process. The crystallization was faster in nucleated samples and the fastest in those with limited entanglements. It was observed that the content of β-crystals decreased with the crystallization temperature. The reduction of the amount of β-forms at high temperatures was not a feature of the nucleant used, but a characteristic future of polymer crystallization at high temperatures.

Analysis according to the Avrami approach showed that at temperatures of 135–140 °C, i.e., when the growth of β-crystals predominated, most nuclei were active in the nucleated, disentangled polymer. The increased efficiency of the introduced nucleant when the macromolecules were disentangled was probably due to the facilitated presence of macromolecules near the active planes of the nucleus, those that were ready to build crystals.

Over a wide temperature range, it was observed that β-phase spherulites grew faster when there were fewer constraints caused by macromolecular entanglements. The effect became more apparent when the measured large difference (5–6 °C) in the equilibrium melting temperature was taken into account. A shift in the range of regime II crystallization of the β-phase in partially disentangled polypropylene was also observed.

All the phenomena described above were caused by the easier movement of macromolecules to the sites of β-crystal grown when the entanglement density of macromolecules was reduced. The greater mobility and freedom of large fragments of macromolecules favored the arrangement of macromolecules near the existing embryo and their transfer into a stable nucleus. The disentangling of macromolecules is even more important during the crystal growth phase, when a straight fragment of the chain is deposited on the surface of the crystal, and it must be able to fold. With less entanglement, the formation of a new layer on the crystal occurs faster, and there are also fewer defects.

## Figures and Tables

**Table 1:** The list of studied materials.

Abbreviation	Macromolecular Entanglement	Content of Nucleant, wt.%
PPi	initial, equilibrium entangled	0
PPi_02	initial, equilibrium entangled	0.2
PPd	partially disentangled	0
PPd_02	partially disentangled	0.2

**Table 2:** Melting and crystallization temperatures as well as melting heats and crystallization heats of the tested polypropylenes.

Sample	I Heating			Cooling		II Heating		
	*T_m_* β [°C]	*T_m_* α [°C]	*H_m_* [J/g]	*T_c_* [°C]	*H_c_* [J/g]	*T_m_* β [°C]	*T_m_* α [°C]	*H_m_* [J/g]
Rate: 10 °C/min								
PPi	-	165 ± 1	90 ± 2	116 ± 1	89 ± 2	147 ± 1	161 ± 1	87 ± 2
PPd	-	165 ± 1	103 ± 2	118 ± 1	89 ± 2	148 ± 1	162 ± 1	95 ± 2
PPi_02	148 ± 1	165 ± 1	93 ± 2	124 ± 1	84 ± 2	151 ± 1	164 ± 1	93 ± 2
PPd_02	146 ± 1	165 ± 1	94 ± 2	125 ± 1	89 ± 2	150 ± 1	162 ± 1	91 ± 2
Rate: 30 °C/min								
PPi_02	158 ± 1		93 ± 2	115 ± 1	84 ± 2	154 ± 1		92 ± 2
PPd_02	158 ± 1		95 ± 2	115 ± 1	92 ± 2	156 ± 1		93 ± 2
Rate: 50 °C/min								
PPi_02	161 ± 1		93 ± 2	111 ± 1	83 ± 2	158 ± 1		90 ± 2
PPd_02	162 ± 1		94 ± 2	112 ± 1	83 ± 2	156 ± 1		88 ± 2

*T_m_* β—melting temperature of the β-phase; *T_m_* α—melting temperature of the α-phase; *T_c_*—crystallization temperature; *H_m_*—heat of melting; *H_c_*—heat of crystallization.

**Table 3:** The values of the *K* coefficient, showing the participation of the β-phase in the entire crystalline phase, determined for the samples crystallized at three different cooling rates.

Material	Cooling Rates during Crystallization		
	10 °C/min	30 °C/min	50 °C/min
PPi	0.02 ± 0.01	0.02 ± 0.01	0.02 ± 0.01
PPd	0.02 ± 0.01	0.05 ± 0.01	0.02 ± 0.01
PPi_02	0.76 ± 0.02	0.81 ± 0.02	0.84 ± 0.03
PPd_02	0.77 ± 0.02	0.81 ± 0.02	0.83 ± 0.02

**Table 4:** β-phase content determined from WAXS measurements.

Sample	*T* = 135 °C	*T* = 140 °C	*T* = 145 °C
PPd_02	0.70 ± 0.02	0.61 ± 0.02	0.09 ± 0.01
PPi_02	0.74 ± 0.02	0.62 ± 0.02	0.19 ± 0.02

**Table 5:** Coefficients from the Avrami equation determined for the samples crystallized at different temperatures.

Sample	135 °C		140 °C		145 °C	
	*n*	*z*	*n*	*z*	*n*	*z*
PPd_02	3.0	3.0 × 10^−2^	3.6	8.1 × 10^−5^	3.8	0.9 × 10^−7^
PPi_0.2	3.4	5.3 × 10^−3^	3.4	4.3 × 10^−5^	3.6	1.3 × 10^−7^
PPd	2.9	5.3 × 10^−4^	3.5	0.8 × 10^−6^		
PPi	3.1	6.8 × 10^−4^	3.7	1.5 × 10^−6^		

**Table 6:** Averaged spherulite growth rates used in spherulite nucleation density calculations. *G′*—averaged spherulites growth rate; *N*—nucleation density.

Sample	T = 135 °C		*T* = 140 °C		*T* = 145 °C	
	*G*′ [µm/min]	*N* [m^−3^]	*G*′ [µm/min]	*N* [m^−3^]	*G*′ [µm/min]	*N* [m^−3^]
PPd_02	5.8	1.5 × 10^14^	1.8	1.4 × 10^13^	0.6	4.2 × 10^11^
PPi_02	5.1	4.0 × 10^13^	1.7	8.8 × 10^12^	0.6	6.0 × 10^11^
PPd	4.9	4.5 × 10^12^	1.5	2.6 × 10^11^		
PPi	4.4	8.0 × 10^12^	1.4	5.5 × 10^11^		

**Table 7:** Growth rate of α-form (*G*_α_) and β-form (*G*_β_) spherulites at selected crystallization temperatures.

Temperature [°C]	*G*_α_ [µm/min]		*G*_β_ [µm/min]	
	PPi_02	PPd_02	PPi_02	PPd_02
126	31.3 ± 0.6	31.3 ± 0.7	45.1 ± 0.7	44.4 ± 0.8
130	12.2 ± 0.5	14.1 ± 0.6	17.2 ± 0.5	18.7 ± 0.6
135	4.4 ± 0.2	4.9 ± 0.4	5.4 ± 0.3	6.3 ± 0.4
140	1.4 ± 0.1	1.5 ± 0.1	1.9 ± 0.1	2.0 ± 0.2
145	0.6 ± 0.1	0.6 ± 0.1	0.6 ± 0.1	0.5 ± 0.1

## Data Availability

Dataset available on request from the corresponding author due to internal rules of the institute.
